# Radiant energy required for infrared neural stimulation

**DOI:** 10.1038/srep13273

**Published:** 2015-08-25

**Authors:** Xiaodong Tan, Suhrud Rajguru, Hunter Young, Nan Xia, Stuart R. Stock, Xianghui Xiao, Claus-Peter Richter

**Affiliations:** 1 Department of Otolaryngology, Northwestern University, 303 E. Chicago Ave, Searle 12-561, Chicago, IL 60611, USA; 2 Department of Biomedical Engineering, University of Miami, Miami FL 33146, USA; 3 Department of Otolaryngology, University of Miami, Miami FL 33136, USA; 4 Key Laboratory of Biorheological Science and Technology, Bioengineering College, Chongqing University, Chongqing 400044, China; 5 Department of Cell and Molecular Biology, Northwestern University Feinberg School of Medicine, Chicago, IL 60611, USA; 6 Advanced Photon Source, Argonne National Laboratory, 9700 South Cass Ave. Argonne, IL 60439 USA; 7 Department of Biomedical Engineering, Northwestern University, 2145 Sheridan Road, Tech E310, Evanston, IL 60208, USA; 8 The Hugh Knowles Center, Department of Communication Sciences and Disorders, Northwestern University, Frances Searle Building, 2240 Campus Drive, Evanston, IL 60208, USA

## Abstract

Infrared neural stimulation (INS) has been proposed as an alternative method to electrical stimulation because of its spatial selective stimulation. Independent of the mechanism for INS, to translate the method into a device it is important to determine the energy for stimulation required at the target structure. Custom-designed, flat and angle polished fibers, were used to deliver the photons. By rotating the angle polished fibers, the orientation of the radiation beam in the cochlea could be changed. INS-evoked compound action potentials and single unit responses in the central nucleus of the inferior colliculus (ICC) were recorded. X-ray computed tomography was used to determine the orientation of the optical fiber. Maximum responses were observed when the radiation beam was directed towards the spiral ganglion neurons (SGNs), whereas little responses were seen when the beam was directed towards the basilar membrane. The radiant exposure required at the SGNs to evoke compound action potentials (CAPs) or ICC responses was on average 18.9 ± 12.2 or 10.3 ± 4.9 mJ/cm^2^, respectively. For cochlear INS it has been debated whether the radiation directly stimulates the SGNs or evokes a photoacoustic effect. The results support the view that a direct interaction between neurons and radiation dominates the response to INS.

Infrared radiation (IR) can be used to directly stimulate nerves, neurons, cardiomyocytes and vestibular hair cells (for a review see Richter and Tan^1^). While the safety and efficacy of infrared neural stimulation (INS) has been demonstrated in various studies, the typical thresholds for INS to elicit neural responses varied significantly^2^,^3^,^4^,^5^,^6^,^7^,^8^. Factors that contribute to these variations are not only the structural and physiological differences of the systems investigated, but include the criteria used to determine a response and the method by which the level of the laser stimulus is reported. Threshold for INS has been defined as the radiant exposure required for a detectable response, including measurements of nerve action potentials, electromyograms, muscle twitches, or other organotypic functional observations. In some publications, the stimulus levels for thresholds are reported in radiant energy^2^, in other studies the radiant exposure is provided^9^,^10^,^11^. The radiant energy is typically measured at the tip of the optical fiber in air and is an objective measure. However, this value does not reflect the radiant energy at the target structure because fluids and tissue between the tip of the optical fiber and the target can absorb and scatter the photons and reduce the incident radiation. To calculate the corresponding energy values at the target for stimulation, the exact distance between the tip of the optical fiber and the target structure and the extinction coefficient are required. The reporting of the radiant exposure is even more complicated. The radiant exposure is, by definition, the ratio of the radiant energy and the corresponding irradiated area (e.g. Welch and van Gemert^12^). In addition to challenges in reporting the energy at the target, it is also difficult to accurately determine the irradiated area or the spot size *in vivo*. The spot size has been either reported as the core diameter of the optical fiber^13^, calculated from the aperture of the optical fiber^5^,^11^,^14^, determined in air and in fluids using the knife-edge technique^13^,^15^, determined using imaging with an infrared sensitive camera^5^, or based on temperature changes reflected by irradiation of thermochromic ink dissolved in agar^16^. Furthermore, any matter between the tip of the optical fiber and the target tissue can scatter the photons and increase the spot size. Thus, it is difficult to provide an accurate estimation of INS parameters for effective and safe stimulation.

In general, INS works through a transient and local heating of the target structure and requires a direct interaction between the infrared radiation and the target tissue^5^,^17^. In excitable cells like neurons, the temperature increase can induce a capacitive current, which depolarizes the cell membrane and induces action potentials. However, heating of a target volume with pulsed infrared lasers also generates a photomechanical or photoacoustic effect, a pressure wave on the target tissue^5^,^18^. In the cochlea, this laser induced pressure wave can activate hair cells. For cochlear stimulation the target structure and the corresponding mechanism of stimulation has yet to be identified. A successful application of INS, specifically for neuroprosthetics like cochlear implants, will require a direct activation of spiral ganglion neurons (SGNs) as well as an optimization of the amount of incident radiation on the target structure. In case the photomechanical or photoacoustic effect is the dominant mechanism of INS in cochlea, the method of stimulation would be of limited benefit in deaf subjects suffering from hair cell loss.

In the present study, we sought to determine the target structure and the radiant energy required for INS in the cochlea. Custom-designed angle polished optical fibers were used for this purpose. The fibers allowed changing the orientation of the radiation beam on and off the spiral ganglion. The responses to INS were determined by the measurements of compound action potentials (CAPs) and neural responses from single units in the central nucleus of the inferior colliculus (ICC). The distances between the optical source and the possible target structures and the orientation of the radiation beam towards the target structure were determined at the conclusion of the experiments from X-ray micro computed tomography (microCT). The spot size at the target structure was calculated from the numerical aperture of the optical fiber and from direct measurements using the knife-edge technique. The radiant energy was measured at the tip of the optical fiber in air and the corresponding energy at the target structure was calculated accounting for the distance and the optical properties of the matter between the fiber and the target structure.

## Results

### Stimulation with flat and angle polished fibers

**Figure 1:**
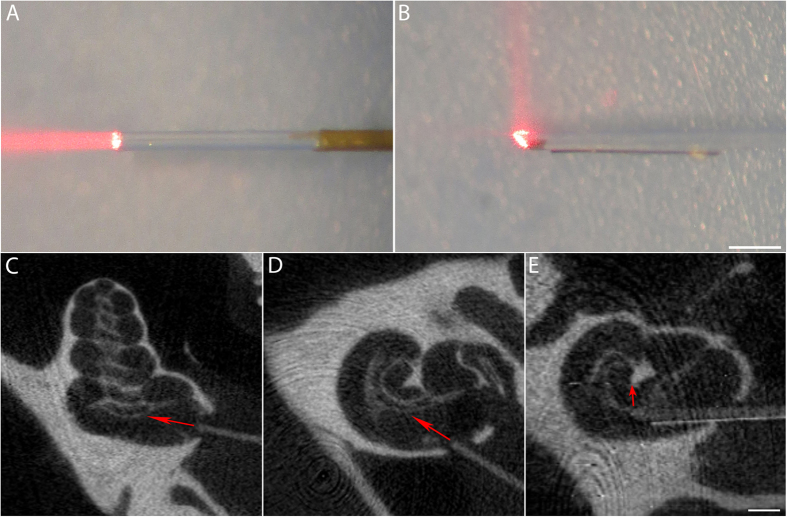
Examples of the two different types of optical fibers used in this study. (**A**,**B**) Flat polished (**A**) and angle polished (**B**) fibers showing the orientation of the beam path. The beam path of the flat polished fiber is along the fiber, while that of the angle polished fiber is perpendicular to the fiber. To localize the tip and orientation of the optical fiber in the microCT images, a 25 μm tungsten wire is glued to the tip of the angle polished fiber opposite to the site the beam exits the fiber. Sometimes a small amount of radiation can be measured straight out of the tip of the optical fiber as shown in the example. This fraction of the energy is usually less than 5% of the total energy of the perpendicular beam. (**C**,**D)** Two reconstructed microCT images from planes perpendicular to each other show the same flat polished fiber inserted into the cochlea. The red arrows show the orientation of the infrared beam, which was towards the modiolus, (**E)** The reconstructed microCT slice shows an angle polished fiber inserted into the scala tympani. Again, the radiation beam is directed onto the spiral ganglion (red arrow). Note that the tungsten wire has much higher contrast than the fiber, which clearly indicates the orientation of the optical fiber. The bars in B and E: 500 μm.

In this study, flat and angle polished optical fibers were used to determine the radiant energy for INS at the SGNs. In Fig. 1, two optical fibers are shown, a flat (Fig. 1A) and an angle polished fiber (Fig. 1B). The beam path was along the optical axis for the flat polished fiber. The beveled surface of the angle polished was coated so that the beam was deflected and exited vertical to the optical fiber. Although not immediately visible, the image also shows that imperfections of the beveled surface might have occurred, which reduced the radiant energy in the beam reflected beam. Calibration before the experiment showed that the imperfection typically accounted for an energy loss of less than 5% of the total radiant energy. Any optical fiber with a “leak” of more than 5% of the total radiant energy was excluded from the study.

Figure 1C,D show the reconstruction of one cochlea after a flat polished fiber was inserted into scala tympani. The light beam was oriented to irradiate the SGNs in Rosenthal’s canal. The planes shown in Fig. 1C,D are perpendicular to each other. Figure 1E shows a slice, which was oriented perpendicular to the modiolus. An angle polished fiber was inserted into scala tympani. Opposite to the site where the beam exits the optical fiber a small section of a 25 μm tungsten wire was attached with acrylic. The placement of the tungsten wire on the optical fiber helped locating the tip of the optical fiber during X-ray imaging and was used to determine the orientation of the beam path from the CT images.

The radiant energy was measured at the tip of the optical fiber in air and evoked responses were recorded for selected energies between 0 and 127 μJ/pulse. From the microCT image, the distance between the tip of the optical fiber and the modiolus could be measured, and the orientation of the beam path could be determined with the help of the tungsten wire.

### Acoustic and optical induced compound action potentials (CAP)

**Figure 2:**
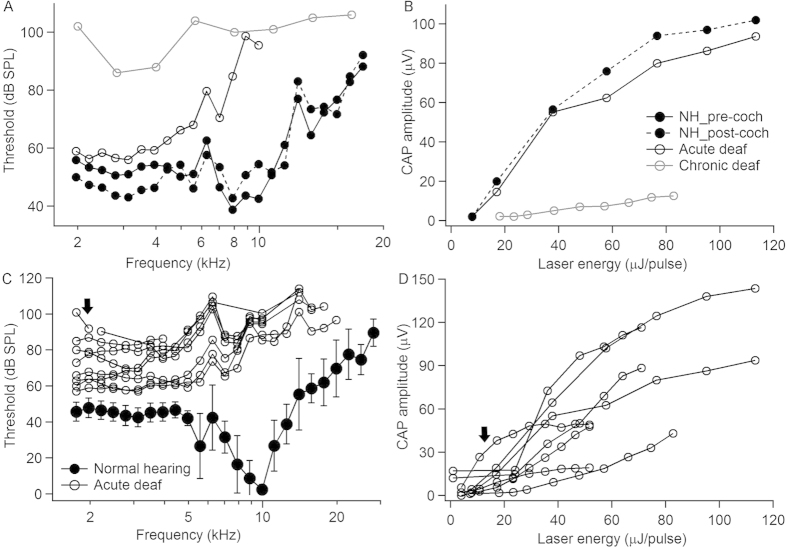
Representative frequency tuning curves and laser input/output curves. (**A**) CAP thresholds to pure tones of a normal hearing (black circles and solid line), an acute deafened (black open circles and dashed line) and a chronic deaf animal (gray trace). (**B**) Laser input/ output curves of the same animals as in panel A. The legends are for both panels A and B. NH_pre-coch: normal hearing animals, pre-cochleostomy; NH_post-coch: normal hearing animal post-cochleostomy. (**C**) Frequency tuning curves of all the acutely deafened animals in this study. The black circles and black solid line represents the averaged threshold of all the animals. Each gray line with circles represents an acutely deafened animal. The arrow shows a particular animal whose acoustic response remains only at lowest frequencies with the threshold higher than 90 dB (re 20 μPa) sound pressure level (SPL). (**D**) Laser input/output of all the animals used in this study after acute deafening. The arrow shows the curve for the same particular animal as indicated in C.

CAP frequency tuning curves to pure tone acoustic stimuli and laser evoked CAP input/output functions were measured to determine cochlear function and the responses to INS. For all stimuli, CAP threshold was defined as the lowest stimulus level at which a response was observed. Representative recordings are shown in Fig. 2. For the normal hearing animal, small threshold changes were observed before (Fig. 2A, solid circles connected by the solid line) and after creating the cochleostomy (Fig. 2A, solid circles connected by the dashed line). The example in Fig. 2A (black open circles) shows that after the neomycin injection into scala tympani, the CAP thresholds at stimulus frequencies higher than 6 kHz were significantly elevated; above 10 kHz no CAP could be evoked acoustically. This was expected from the location of the neomycin injection, which was at the base of the cochlea. The CAP threshold for acoustic clicks was elevated by 55 dB in this animal, from 35 dB (re 20 μPa) sound pressure level (SPL) before deafening to 90 dB SPL after deafening. Similar results were obtained in a chronic deaf animal. For this example, the hearing thresholds at any frequency were over 85 dB SPL (Fig. 2A, gray open circles). These two animals, whose cochleae were damaged with neomycin showed robust CAPs in response to INS (Fig. 2B). Interestingly, there was no threshold shift to INS before and after acute deafening. In the chronic deaf animal, the threshold shift was about 6 dB (threshold energy: ~37 μJ/pulse) compared to that of the normal hearing animals (~18 μJ/pulse). Since residual low frequency hearing remained in the acutely deafened animals, one might argue that a photomechanical or photoacoustic event stimulated the low frequency section, the apex of the cochlea. Note, our data showed that the CAP response was also measured in a chronic deaf animal with profound hearing loss for the entire frequency range. The latter results suggest that the photomechanical or photoacoustic event was not likely the dominant mechanism for stimulation.

Figure 2C shows the frequency tuning curves for all of the animals investigated. The dark trace provides the average values and corresponding standard deviations for the normal hearing animals. The curves with circles represent the individual animals. Clearly the threshold was elevated to different degrees after the deafening procedure. Significant elevation in CAP threshold was always observed at frequencies higher than 8 kHz, and the responses to acoustic stimuli above 20 kHz were absent in all animals (no response up to 110 dB SPL). Again, this is consistent with the location of the neomycin injection at the cochlear base. Figure 2D shows the laser input/output of the angle polished fibers for all the animals used in this study. Although the threshold for acoustic stimulations changed drastically in these animals, the threshold for INS has little or no change after acute deafening (Fig. 2D). In one particular animal, the acoustic response could only be detected at the two lowest frequencies tested at sound levels higher than 90 dB SPL (Fig. 2C, the arrow), while robust laser responses were still elicited (Fig. 2D, the arrow).

### Location and orientation specific changes with the angle polished fibers

#### CAP amplitude changes with the location

**Figure 3:**
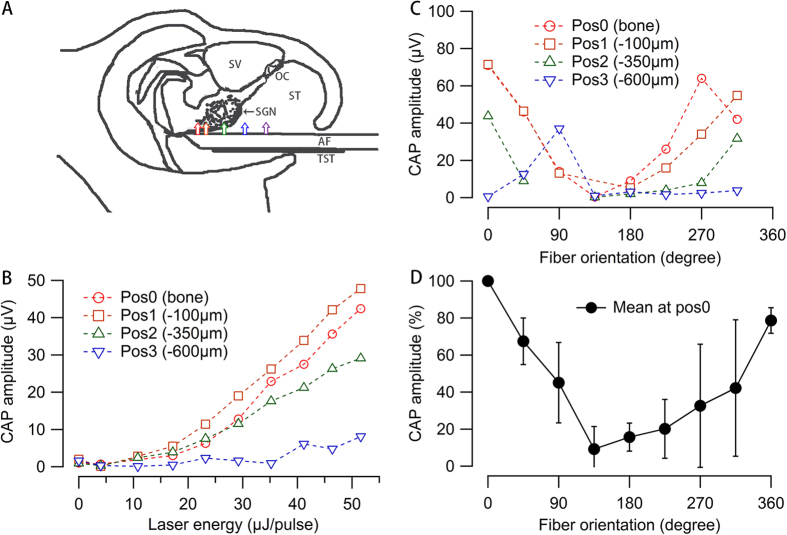
CAPs evoked with angle polished fibers. (**A**) Sketch of Fig. 1E showing the locations of INS in the cochlea along the beam path while the optical fiber were retracted from the modiolus. The angle polished optical fiber was inserted along the scala tympani of the basal turn until it contacted the bony structure of modiolus. The fiber was then rotated until the maximum CAP amplitude was obtained. While maintaining the same orientation, the optical fiber was retracted from scala tympani using a micromanipulator. The CAP was measured at locations of the tip of the optical fiber marked by the colored arrows, namely 0, 100, 350, 600 and 850 μm away from the modiolus in consequence. The colored arrows also indicate the orientation of the beam so that the radiation target structures can be inferred. AF: angle polished optical fiber; OC: organ of Corti; SGN: spiral ganglion neuron; ST: scala tympani; SV: scala vestibuli; TST: tungsten wire. (**B**) Input-output curves of the 4 marked positions as in panel A showing CAP amplitude versus radiant energy profiles (amplitude-level curves). No CAP response could be measured at position 850 μm. Note that at all the positions measured, CAP amplitude always increased with laser energy. (**C**) Orientation-specific CAP changes in the 4 marked positions as in panel A. The orientation of the beam towards the modiolus is marked as 0° and increased counterclockwise. The energy level was fixed at 82 μJ/ pulse. Again, no CAP was evoked in position 4. (**D**) The average orientation-specific CAP changes across animals at position 0. The data were normalized by the maximum responses at the orientation 0°.

The angle polished fiber was inserted into the cochlea until contact was made with the modiolus and was rotated until a maximum CAP amplitude was obtained. While maintaining the same orientation, the optical fiber was retracted using a micromanipulator and CAP amplitude was measured at different locations along the track. A typical sequence of measurements, which was obtained in one animal, is shown in Fig. 3A. The CAP amplitudes to INS were determined at locations 0, 100, 350, 600, 850 μm away from the modiolus. From the sketch of a reconstructed micro-CT slice, the target structures at the 5 locations were identified. The orientation of the laser beam was indicated by the arrows so that the radiation target structures could be inferred from the sketch. At each marked position, CAP responses to different energy levels were measured while the orientation of the optical fiber was kept consistent towards the modiolus. As shown in Fig. 3B, the CAP amplitude was maximum while the entire beam included SGNs (0–100 μm), decrease when the beam partially off the target (350 μm), and was minimum when completely off the SGNs (600 μm). No response was detected when the beam was directed towards the basilar membrane (850 μm). The input-output curves showed consistent changes at different positions.

#### Changes with the orientation of the radiation beam

At marked locations in Fig. 3A, CAP responses at different fiber orientations were measured and plotted to determine the change of the CAP amplitude with the beam orientation (Fig. 3C) at the radiant energy of 82 μJ/pulse. The radiant energy was selected to evoke a response well above stimulation threshold. Moreover the energy was below the level above which saturation in the input output function was observed. The beam orientation was initially set towards the SGNs and marked 0°. After the initial measurements, CAP responses were measured every 45° counterclockwise until a full turn was measured. As shown in Fig. 3C, the maximum CAP amplitude was always observed at or close to 0°, when the radiation beam was oriented directly toward the Rosenthal’s canal, where the SGNs were located. The CAP amplitude decreased gradually while the beam was moved off the SGNs and was minimum at 180°, where the beam was directly opposite to the SGNs. Large variations were seen at 225°-315°, where the beam orientation was directed to the basilar membrane. After a full turn (360°), the orientation of the laser beam was back to the original location, and the maximum CAP amplitude was measured again. Same full turn measurements were conducted in 12 animals at position 0 μm to the modiolus and the change of CAP amplitude was consistent in all the animals (Fig. 3D). In some cases, the CAP response did not completely disappear even when the fiber was opposite to the spiral ganglion. We should mention that in some animals, the CAP amplitude decreased significantly after a full turn. This accounted for about 20% decrease at 360° in the plot (p = 0.03, paired t-test). A possible explanation could be a local cochlear damage of the cochlea and SGNs caused by breaking of Rosenthal’s canal during the rotation and repositioning of the optical fiber. Nevertheless, the maximum response was always at the same orientation, before and after the full rotation of the angle polished optical fiber.

#### Selective activation and orientation-dependence of ICC responses

**Figure 4:**
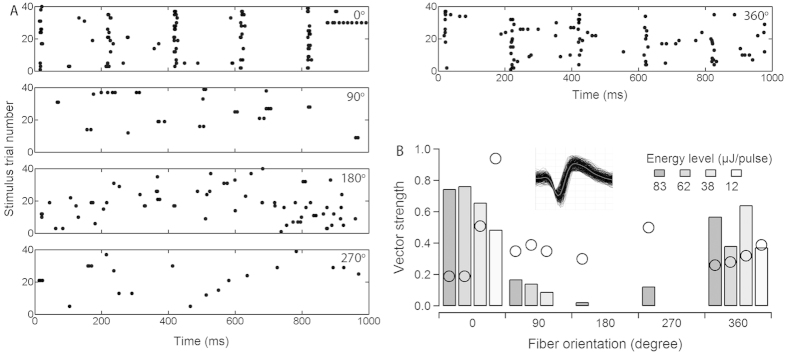
Responses of single units from the ICC obtained during INS. (**A**) A representative example of single unit responses to a continuous 5 Hz INS pulse trains at orientations of 0, 90, 180, 270 and 360° is shown in the raster plots. Each dot represents an action potential. Note that the phase-locked responses at ~24 ms following stimulus presentation to 5 Hz INS pulse trains were only observed in the initial orientation (0°) and recurred at the same orientation (360°) after a full turn of rotation. (**B**) Vector strengths in response to INS at different energy levels and orientations. When the vector strength is higher than the Rayleigh criteria (open circles superimposed on each column), the response is considered significantly phase-locked (P < 0.001). The inset shows the uniformity of all the action potential waveforms recorded. The threshold stimulation energy of the single units was 12.4 μJ/pulse.

Since the CAP is the summation of the responses from many neurons along the cochlea it is not clear whether the response comes right from the target structure. For example, photoacoustic events caused by the infrared irradiation may activate large areas of the cochlea. To study local responses to INS along the cochlea, recordings in the ICC were conducted with multichannel electrodes, which were inserted in the ICC across frequency laminae. Multi-unit responses might be recorded at each electrode contact of the multichannel electrode array^19^. Therefore, an offline sorting procedure was performed for each recording to identify single units. Responses from well-isolated single units should have more precise orientation-dependence. An example is shown in Fig. 4A. The 5 panels in Fig. 4A show the responses of the single units to INS at orientations 0, 90, 180, 270 and 360°, respectively. Phase-locked responses to 5 Hz trains of laser pulses were only observed in the initial orientation (0°) and recurred at the same orientation (360°) after a full turn rotation of the angle-polished fiber. Phase-locked responses were determined by calculating the vector strength from single units in the ICC with well-isolated action potential waveforms such as the one shown in the inset of Fig. 4B. We used the Rayleigh test to determine the statistical significance of phase-locked firing (Fig. 4B). For this single unit, the threshold radiant energy required for stimulation in the ICC was 12.4 μJ/pulse.

**Figure 5:**
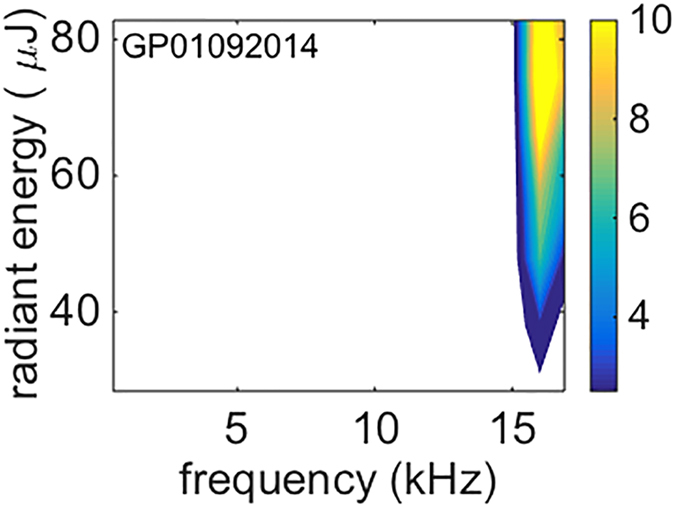
The spatial response profile of a single unit response in the ICC during INS. Data were acquired in a normal hearing animal and recorded with a 16-channel electrode. INS was delivered by an angle-polished optical fiber. The best frequency at each channel was determined using pure tone stimuli with different frequencies and stimulus levels before the insertion of the optical fiber into scala tympani. The single unit responses were only detected in 2 channels with best frequencies of 16 kHz and 17 kHz, respectively. Results were obtained at the orientation where the maximum response was evoked (marked as 0° in the text). Similar responses were observed in a ±45° range, and no response was detected for any other orientations. The color code: spike counts per 100 ms.

**Figure 6:**
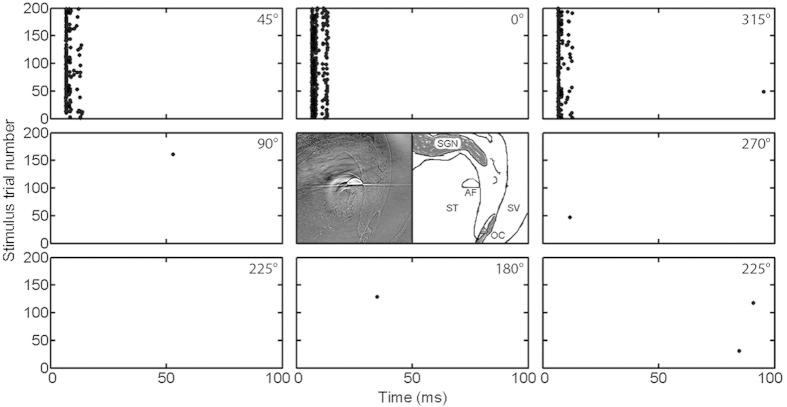
ICC single unit responses to different INS orientations in a normal hearing animal combined with imaging with synchrotron radiation. The single unit responded to acoustic stimulation as well, with a best frequency at 16 kHz. (**Central panel**) A reconstructed slice perpendicular to the fiber axis and its sketch. The angle polished fiber (AF, the bright semi-circle in the center) was fixed with dental acrylic in the cochlea at the initial position (0°) for imaging with synchrotron radiation. Notice that the fiber was located between the spiral ganglion neurons (SGN) and the organ of Corti (OC) facing the SGNs. ST: Scala tympani; SV: Scala vestibuli. (**Surrounding panels**) Single unit responses to different orientations of the INS changing in step of 45°, as indicated in the upright corner of each panel, shown for 200 stimulus trials. This ICC unit showed phase-locked responses at ~7 ms following stimulus presentation. Only the action potentials detected in the first 100 ms following the stimulus were included in the raster plot.

To further identify the target structure for INS, single unit recordings in the ICC were combined with synchrotron microCT imaging in 3 normal hearing animals. The possible involvement of hair cells in the response to INS should be more obvious in normal hearing animals. The selective stimulation of INS in the cochlea is well-represented in the spatial activation profile in ICC. An example is shown in Fig. 5. The best frequency at each electrode contact was measured and identified as marked on the x-axis. Although the acoustic stimulation evoked responses at most electrode contacts, INS evoked single unit responses occurred at the deepest 2 channels with best frequencies of 16 and 17 kHz, respectively. This is consistent with the location of the INS, which was at the basal cochlea. Rotation of the angle-polished fiber showed that the INS induced single unit responses were only observed in a ±45° range. At the conclusion of the experiment, the angle polished fiber was fixed in the cochlea using dental acrylic at the orientation at which the maximum responses were induced (0°). Figure 6 shows a well-isolated single unit from the recordings combined with the X-ray imaging. A section from the tomographic reconstruction of the cochlea and its corresponding sketch are shown in the central panel of Fig. 6. The beam path of the fiber is vertical to the beveled surface (the bottom flat line of the semi-circle in the middle) and up toward the SGN as indicated in the sketch. The single unit responses for 0–360° orientations in increments of 45° are shown in the surrounding panels. Robust responses were observed when the beam was oriented towards the SGNs, between 45° and −45° (315°). The location of the hair cells was approximately opposite to SGNs (~180°). Response to INS was not observed when the infrared beam was directed towards the hair cells.

### Radiant energy at target structure

#### Radiant energy to reach threshold for stimulation

**Figure 7:**
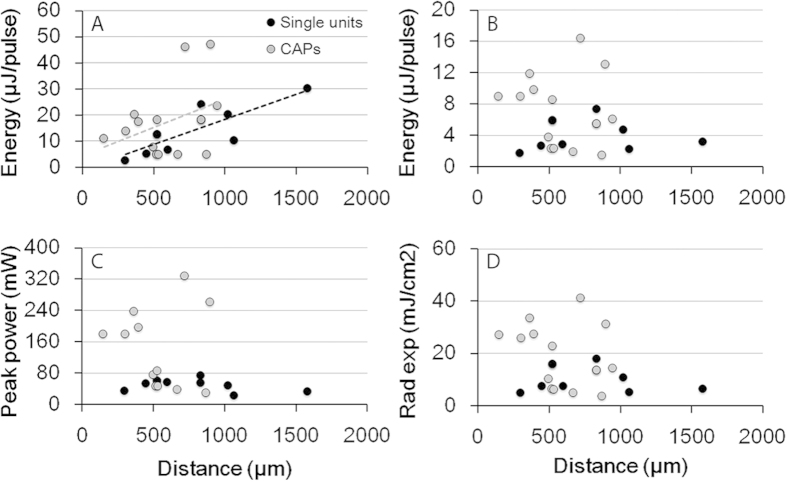
(**A**) Threshold radiant energy for stimulation of spiral ganglion neurons was measured at the tip of the optical fiber from ICC response profiles (filled black circles) and CAP measurements (black circles). (**B**) The corresponding corrected energy values at the modiolus. (**C)** The corresponding peak power for stimulation threshold at the modiolus. (**D**) The corresponding radiant exposure.

The radiant energy required for stimulation was determined from the experiments in which CAP amplitudes or ICC single unit responses were measured. Threshold for INS of single units was defined as the radiant energy required to evoke significant phase-locked firing. The energy at the tip of the optical fiber was on average 14.1 ± 8.1 μJ/pulse for ICC for single unit responses and 17.2 ± 13.9 μJ/pulse for CAP response, respectively (Fig. 7A). The variation of the radiant energy at the tip of the optical fiber to reach stimulation threshold was large with a range of 4.8 to 47 μJ/pulse. These thresholds were all obtained when the orientation of the radiation was towards the modiolus (the orientation of optical fiber at 0 degree).

The threshold energy required for producing a response increased with the distance between the tip of optical fiber and the modiolus in both CAP and ICC levels (Fig. 7A. r^2^ = 0.67). The radiant energy at the target structure is influenced by the radiation spot size of the laser, which can be calculated by the numerical aperture of the fiber and by the distance between the tip of the optical fiber and the neural structure. Therefore, we measured this distance using the reconstructed microCT images. This distance varied between 150 and 950 μm (589.6 ± 243.8 μm, n = 14). The spot size at the target structure was calculated accordingly as has been presented in a previous publication^13^ (see Methods). The calculated spot size, which was on average 0.041 ± 0.005 mm^2^, was then used to estimate the energy at the target structure to reach response threshold. After correcting for the distance between the tip of the optical fiber and the modiolus, the radiant energy on target was between 1.4 and 16.4 μJ/pulse, on average 4.1 ± 1.9 μJ/pulse for ICC single units and 7.2 ± 4.7 μJ/pulse for CAP responses, respectively (Fig. 7B). The corresponding averaged peak power was 48.4 ± 15.5 and 128.9 ± 98.2 mW, and the radiant exposure was 10.3 ± 4.9 and 18.9 ± 12.2 mJ/cm^2^, for the ICC and CAP responses, respectively (Fig. 7C,D).

## Discussion

In this study we provide evidence for a direct stimulation of SGNs with INS combining the physiological responses with X-ray tomography using angle-polished fibers. We also determined the threshold energy on target for INS in cochlea, which is 4.1 ± 1.9 μJ/pulse at single unit level.

### CAP versus ICC responses

Our results suggest that the threshold radiant exposures are in general larger for CAP amplitude measurements than for recordings from the ICC, 18.9 versus 10.3 mJ/cm^2^ (Fig. 7). The difference may be explained by the parameter that has been used to determine the threshold. The CAP constitutes a synchronized response of a population of neurons to the irradiation^20^,^21^. In contrast to the CAP responses, the ICC responses reflect the neural activity of single or a small number of neurons. The stimulus level required to reach threshold of a single auditory nerve fiber or neuron cluster in the ICC will be less than that required to evoke a compound nerve response. In the literature it has been well documented that thresholds for single auditory nerve fiber recordings are lower when compared to compound action potential measurements or behavior of the animals (e.g. Dallos, Harris^22^). Thresholds for single fiber recordings correlate well with thresholds obtained in experiments that used animal behavior to assess hearing thresholds. Based on these results, it is fair to select threshold radiant exposures obtained with the ICC recordings as the stimulation threshold for INS. To verify this assumption, behavioral responses need to be recorded and compared with CAP and ICC measurements.

Figure 7 shows a large variation for the CAP thresholds for INS. Since the results have been corrected for the different distances of the optical fiber from the target structure, the findings suggest that the distance does not likely contribute to the variation. However, depending upon the orientation of the optical fiber the infrared beam may irradiate a different volume of the spiral ganglion. For example, if the optical fiber is positioned perpendicular to Rosenthal’s canal, the number of neurons in the beam path will be smaller. In comparison, a larger number of SGNs will be irradiated when the orientation of the fiber results in a beam path along Rosenthal’s canal.

### Spot size considerations

Results on INS have been published over the last decade by several groups (for a review see e.g. Goyal *et al*.^23^ or Richter and Tan^1^). The criterion by which threshold for stimulation is defined has varied considerably among previous studies. While some use direct electrical measurements from the nerve, others record electrical responses from the target structure, or visually determine threshold by the effect of stimulating the nerve, such as muscle twitch^14^, pacing of the embryonic heart^7^, or cavernous pressure^3^,^24^,^25^. Moreover, the stimulus level is reported either in radiant energy per pulse, average power, peak power, or radiant exposure. While radiant energy per pulse, average and peak power can be directly measured at the tip of the optical fiber in air, the reporting of the radiant exposure requires the accurate determination of the spot size. The spot size can be determined with the knife-edge technique as the width of the resulting energy profile at half maximum of the energy^13^,^15^, as the area of the optical fiber^13^, or has been calculated by using the numerical aperture and the distance of the target from the tip of the optical fiber^5^,^11^,^14^. In two different studies either a camera which is sensitive to infrared radiation^11^ or thermochromic ink^16^ was used to determine the spot size of the laser beam at the target structure. Each of the approaches described above provides slightly different values for the spot size and makes a direct comparison of threshold radiant exposures among the studies difficult. Radiant energy or power at the tip of the optical fiber in air can be reported reliably and should be provided for each study. Furthermore, it is more than desirable that estimates of radiant energy and/or radiant exposure are reported at the target structure. For example, in this study we report the radiant energy per pulse at the tip of the optical fiber in air. The values are corrected based on the absorption and scattering that might occur along the beam path (between the tip of the optical fiber and the modiolus). The spot size is calculated using the optical properties of the different media and the optical properties of the fiber that is used to deliver the radiation.

### INS versus electrical stimulation: power considerations

Current neural prostheses use electrical current to stimulate neurons. It has been demonstrated that stimulation threshold correlates with the charge delivered. For example, the charge per phase that is required to stimulate the auditory nerve is ~1 nC/phase (e.g. Clark^26^). This corresponds to 10 μA for a 100 μs/phase. With an electrode contact resistance of about 8 kΩ the corresponding electrical energy is 0.8 μJ/phase. For INS, the minimum power that was required to reach threshold was about 2.6 (ICC) to 4.5 (CAP) times larger. For the realization of an optical neural prosthesis, optimization in delivery of the infrared radiation will be clearly necessary. Three general approaches may be apparent: an optical fiber bundle, light guides, and small laser sources such as Vertical Cavity Surface-Emitting Lasers (VCSELs). Optical fibers are stiff and their insertion depth is limited given that the radius of curvature in the cochlea is small^27^. Further challenges include the optimization of the fiber size and associated coupling losses and losses that occur through bending of the fiber. Moreover, it will be challenging to pre-determine the orientation of each of the angle polished fibers towards the spiral ganglion. Optical light guides have not been produced and tested for the given long-infrared wavelength (λ = 1862 nm). Again the challenge will be that the light guides are small enough to allow the fabrication of an array of “light sources” but sufficiently large to deliver the required energies and optical spot size to stimulate neurons. Small laser sources like VCSELs can be placed similar to electrical contacts of a contemporary electrical cochlear implant electrode. The stimulation of more apical sites along the cochlea, i.e., the low frequency part, could also be achieved with this technique. The challenge with this approach will be the efficiency of converting electrical energy into radiant energy. For small light sources the efficiency is presently only about 30%. The remaining energy is converted into heat and can damage cochlear structures. Thus, careful consideration of the temperature load of the implantable device and improvements in the design of these devices are required.

### Mechanism of INS

The mechanism of INS, in particular the contributions of the pressure which is generated through local heating through the absorption of the laser pulses, has recently been discussed in detail in a separate paper by Young *et al*., Neurophotonics. 2015 Apr;2(2):025002. doi: 10.1117/1.NPh.2.2.025002. Epub 2015 May 18. Transient heating of the neural tissue through the absorption of photons constitutes the one step of the mechanism of INS everybody can agree to^1^,^28^. How the heating is converted into an action potential is not clear today. Several possibilities have been discussed and include the generation of a depolarizing capacitive current^17^,^29^,^30^, the activation of temperature sensitive ion channels such as the Transient Receptor Potential (TRP) channels^31^,^32^,^33^,^34^, increase of intracellular calcium concentration^35^,^36^,^37^, and the generation of a pressure wave that mechanically stimulate the nerves^38^,^39^,^40^.

In particular, the experiments by Schultz *et al*. suggest that a pressure wave is the sole mechanism by which the cochlea is stimulated with INS pulses in nanoseconds^38^. Following the injection of large amounts of neomycin into scala media, responses to auditory and optical stimuli disappeared. However, responses to monopolar electrical stimulation were present but reduced in amplitude. From the results it was concluded that cochlear INS is the result of a pressure wave by the laser. If indeed INS is effective solely through a pressure wave during tissue irradiation, it does not explain the results of the present study. Angle polished fibers were used to determine the dependence of the responses from the orientation of the beam path. As can be seen from the experiments documented in Fig. 5, neural stimulation only occurred while the SGNs were in the beam path. On the other hand, while the laser was pointed towards the organ of Corti or the basilar membrane no or only small responses could be recorded. Furthermore, orientation dependence has been also shown at the single unit level (Figs 4 and 5).

Recent work by Ren *et al*. showed a photomechanical vibration of basilar membrane when it was irradiated directly with a 930 nm laser^41^. The irradiation resulted in a local initial passive mechanical deflection of the basilar membrane. In pristine cochleae, this initial basilar membrane movement was followed by a ringing response, which was evoked by the cochlear amplifier. Activation of the cochlear amplifier was not local but could also be seen away from the stimulation site in pristine cochleae. In damaged cochleae, the photomechanical events only occurred locally at the irradiation site and the active ringing disappeared. This has been documented by measurement on postmortem cochleae in their work^41^ and verified by personal communications with Dr. Ren. Our stimulation paradigm was different from theirs in that the incidence angle of laser irradiation to the organ of Corti was much smaller (Fig. 6). This could result in a pressure wave largely parallel to the basilar membrane and thus reduce the direct photomechanical effect. The orientation dependent INS responses indicate that the evoked CAP and ICC neural responses in our study are mainly through the direct stimulation of SGNs. On the other hand, our data did not exclude the involvement of the basilar membrane. In the orientation specific CAP amplitude changes (Fig. 3C,D), the variations at 225°–315° were huge compared to other orientations. This is where the organ of Corti locates as shown in the microCT image in Fig. 6 and suggesting the contributions from the basilar membrane is possible in normal hearing or residual hearing animals.

While parts of the mechanism for INS have been identified^17^,^35^,^39^,^41^, a comprehensive understanding of the mechanism of INS is still missing and further research is required. Our results indicate a direct stimulation of SGNs with INS, which is strongly supported by the orientation specific responses at the single unit level with the side-firing fiber. This matches perfectly with our early work showing that the stimulation in the cochlea was determined by the beam orientation of the optical fiber^16^. In some animals the CAP responses could still be detected at the orientation opposite to spiral ganglion. The possible reason is that the spiral ganglion was still partially located in the beam path because of the 3D spiral structure, even though the laser is pointed in a direction that is 180° off the angle for the maximum response. Stimulation of remaining hair cells by either photothermal or photomechanical/photoacoustic effect through direct irradiation could also be a possible source of the response. Another possibility relates to the fact that photons scattered from the bony wall, which could also result in a neural response.

## Methods

Pigmented guinea pigs (200–800 g) of either sex were used in the experiments. Care and use of animals were carried out in accordance with the NIH Guide for the Care and Use of Laboratory Animals and were approved by the Animal Care and Use Committee of Northwestern University.

### Surgical access to the cochlea and insertion of the optical fiber

Surgery and data acquisition were similar to those described in detail elsewhere^2^. The animals were initially injected with urethane (1.3 g/kg i.p.) and the anesthesia was maintained by supplements of ketamine (44 mg/kg) and xylazine (5 mg/kg). The depth of the anesthesia was assessed every 15 minutes by a paw withdrawal reflex. The core body temperature was maintained at 38 °C. A tracheotomy was made and a plastic tube connecting to an anesthesia system (Hallowell EMC, Pittsfield, MA) was secured into the trachea. The animals were ventilated on oxygen throughout the length of the experiment. The head of the animal was mounted in a stereotactic head holder (Stoelting, Kiel, WI). A c-shaped skin incision was made behind the left ear lobe. The cervicoauricular muscles were removed and the cartilaginous outer ear canal was exposed and cut. The left bulla was exposed and opened approximately 2 × 3 mm with a motorized drill (World Precision Instruments, Sarasota, FL). A cochleostomy was created in the basal turn with a 0.5 mm Buckingham footplate hand drill (Richards Manufacturing Co., Memphis, TN). A flat-polished or angle-polished optical fiber mounted to a micromanipulator (MHW103, Narishige, Tokyo, Japan) was inserted through the cochleostomy (Fig. 1). The numerical aperture of the polished fibers was 0.22. The acceptance angle was previously determined with the knife-edge method and was about 1.0° in fluids^2^,^13^. The flat polished fibers were inserted perpendicular to the modiolus. The angle polished fibers were inserted along the cochlear turn until contact was made with the bony cochlear wall. The orientation of the fiber, the radiation beam was initially directed towards the modiolus. Subsequently the fiber was systematically rotated until a maximum response was measured. To increase the contrast of the fiber in the microCT image afterwards, a 25 μm tungsten wire was glued close to the tip of the angle polished fiber by the beveled surface side (Fig. 1B,E).

### Infrared neural stimulation

Cochlear stimulation was achieved with diode lasers (Lockheed Martin Aculight Corp., Bothell, WA) coupled to the optical fibers (P200-5-VIS-NIR, Ocean Optics Inc., Dunedin, FL) with core diameters of 200 μm (Fig. 1A,B). The radiation wavelength was 1862 nm and the pulse duration was 50 or 100 μs. The radiant energy at the tip of the optical fiber was calibrated in air prior to the experiments and was between 0 and 127 μJ/pulse. Pulses were presented either as single events at 2–5 Hz, or at a 250 Hz repetition rate in pulse trains of 300 ms duration. For each data point 10 pulse trains were averaged.

The energy *E* at the modiolus was calculated with the following equation: *E* *=* *E*_*o*_ * *e*^−μ*d*^, where *E*_*0*_ denotes the energy per pulse at the tip of the optical fiber, *μ* the extinction coefficient, and *d* the distance between the optical fiber and the modiolus that was determined from the microCT scans (see below). From the radiant energy the peak power was calculated by dividing the *E* by the pulse duration. The radiant exposure was calculated by dividing *E* by the spot size. The spot size was calculated by using the core diameter of the optical fiber *d*_*fiber*_, and the spread of the radiation in water as determined in previous experiments *r* = *d* × tan *α*^13^, where *α* is the angle by which the radiation beams spreads. The radius *R* for the spot was calculated as 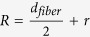.

### Compound action potential (CAP) measurements

CAPs were measured with a silver ball electrode at the round window and a reference electrode in the tissue near the neck. Threshold was defined as a CAP that was visible above the noise floor of the recordings, typically between 3–10 μV. CAPs to acoustic stimuli were measured before and after creating the cochleostomy and after neomycin application. For the angle polished fiber, threshold energy and response amplitude was determined for the initial placements of the optical fiber. An energy-versus-CAP amplitude contour (laser input/output curve) was recorded for at least 10 different radiant energy levels. Next, the fiber was rotated by 45° counterclockwise and the procedure was repeated until a full rotation (360°) was completed. The optical fiber was then rotated to the pointing angle at which the largest CAP amplitude was measured and was retracted in steps of 50 μm. CAP amplitudes in response to a fixed radiant energy (typically 40 μJ/pulse) was measured at each step.

### ICC recording with multichannel electrodes

A multichannel penetrating electrode array (A1 × 16–5 mm-100–177, NeuroNexus Technologies, Ann Arbor, MI) was used for ICC recordings^2^. Each array had 16 recording sites (177 μm^2^/site) along a single shank at center-to-center intervals of 100 μm. To access the ICC, a 5 × 5-mm opening was made in the right parietal bone. The multichannel electrode mounted on a 3D-micromanipulator (Stoelting, Kiel, WI) was advanced through an incision in the dura mater and through the occipital cortex into the ICC. The electrode was inserted into the ICC on a dorsolateral to ventromedial trajectory at approximately 45° off the parasagittal plane, approximately orthogonal to its isofrequency laminae^2^,^42^,^43^. Proper placement of the electrode was determined when neural responses from the distal contact of the array could be stimulated with a tone pip between 16–25 kHz and single unit responses to INS were recorded.

In most cases, the ICC recording electrode array was placed prior to creating the cochleostomy because the procedure could result in an elevation of the CAP thresholds and lead to a poor correlation of the ICC location to the best acoustic frequency.

### Deafening of the animals

Measurements were conducted in normal hearing and deaf animals (Fig. 2). Acute deafening was achieved by injecting neomycin (20 mM, 50 μL in Ringer’s Lactate) into scala tympani through the cochleostomy. Deafening was not always complete and remaining cochlear function was verified by determining acoustic CAP thresholds pre- and post-deafening. Animals were considered to be deaf when the CAP threshold of a click response was elevated for at least 40 dB and higher than 80 dB SPL.

The orientation specific changes of CAPs induced by INS were also verified in a chronic deaf animal. For chronic deafening, at least 4 weeks before the experiments, neomycin (25 mM, 250 μL) was injected in the bulla through tympanic membrane. The pure tone CAP thresholds were over 80 dB SPL (Fig. 2A,B) and the click CAP threshold of the animal was 94 dB SPL.

### The beam path determined with microCT and synchrotron radiation

Before insertion, a 25 μm tungsten wire was attached at the tip of the optical fiber with Loctite super glue (Henkel Corporation, Rocky Hill, CT), opposite to the site where the radiation beam exited the optical fiber (Fig. 1B,E). The tungsten wire has a high contrast in the X-ray imaging and can be easily identified. At the end of each experiment, the optical fiber was left in the cochlea at the position of the maximum response. The fluid in the cochlea was wicked away and replaced by a viscous solution of dental acrylic. After the acrylic had cured, the animals were euthanized with an overdose of sodium pentobarbital (Euthasol^®^) and followed by cardiac perfusion with 2.5% glutaraldehyde and 1.5% paraformaldehyde (pH 7.4) in Ringer’s Lactate (RL). After fixation, the animals were decapitated and the bullae were harvested with the optical fiber in place. The specimens were imaged using microCT (Scanco Medical, Wayne, PA) and synchrotron radiation at 2-BM at the Advanced Photon Source (APS), Argonne National Laboratory. From the tomographic reconstruction, a plane across the center of the optical fiber and the tungsten wire and the target structure was selected. The distance between the optical fiber and the target structure was then measured in this plan.

### Data analysis and statistics

The phase-locked firing of the ICC single units was evaluated by calculating the vector strength^44^,^45^,^46^. Rayleigh’s test for circular uniformity^47^ was used to test for statistical significance at a level of P < 0.001. The lowest energy at which the vector strength was higher than the significance level of Rayleigh’s test was defined as the threshold energy of the unit (refer to Fig. 4B and the Results).

## Additional Information

**How to cite this article**: Tan, X. *et al*. Radiant energy required for infrared neural stimulation. *Sci. Rep*. **5**, 13273; doi: 10.1038/srep13273 (2015).
